# Correction: Pediatric Diabetic Ketoacidosis (PDKA) among newly diagnosed diabetic patients at Dilla University Hospital, Dilla, Ethiopia: Prevalence and predictors

**DOI:** 10.1371/journal.pone.0330202

**Published:** 2025-08-11

**Authors:** Dinberu Oyamo Oromo, Asmare Melka Melaku

Asmare Melka Melaku should be included in the byline. Asmare Melka Melaku should be listed as the second author, and his affiliation is: Department of Pediatrics and Child Health, College of Health Sciences, Dilla University, Dilla, Ethiopia. The contributions of this author are as follows: Conceptualization, Formal Analysis, Investigation and Methodology.

The correct citation is: Oromo DO, Melaku AM (2025) Pediatric Diabetic Ketoacidosis (PDKA) among newly diagnosed diabetic patients at Dilla University Hospital, Dilla, Ethiopia: Prevalence and predictors. PLoS ONE 20(1): e0314433. https://doi.org/10.1371/journal.pone.0314433.
