# Supplementary material for: Pediatric Diabetic Ketoacidosis (PDKA) among newly diagnosed diabetic patients at Dilla University Hospital, Dilla, Ethiopia: Prevalence and predictors
Source: PLoS One. 2025 Jan 30;20(1):e0314433. doi: 10.1371/journal.pone.0314433 (PMC11781625; doi:10.1371/journal.pone.0314433)
Supplement: S1 File — (DOCX) [file pone.0314433.s001.docx]

DATASET ACTIVATE DataSet1.

SAVE OUTFILE='C:\Users\user\Documents\asmare research_1.sav'

/COMPRESSED.

FREQUENCIES VARIABLES=age

/BARCHART FREQ

/ORDER=ANALYSIS.

**Frequencies**

| **Notes** | | |
| --- | --- | --- |
| Output Created | | 29-DEC-2022 14:07:52 |
| Comments | |  |
| Input | Data | C:\Users\user\Documents\asmare research_1.sav |
|  | Active Dataset | DataSet1 |
|  | Filter | <none> |
|  | Weight | <none> |
|  | Split File | <none> |
|  | N of Rows in Working Data File | 61 |
| Missing Value Handling | Definition of Missing | User-defined missing values are treated as missing. |
|  | Cases Used | Statistics are based on all cases with valid data. |
| Syntax | | FREQUENCIES VARIABLES=age  /BARCHART FREQ  /ORDER=ANALYSIS. |
| Resources | Processor Time | 00:00:00.17 |
|  | Elapsed Time | 00:00:00.17 |

| **Statistics** | | |
| --- | --- | --- |
| Age of child | | |
| N | Valid | 61 |
|  | Missing | 0 |

| **Age of child** | | | | | |
| --- | --- | --- | --- | --- | --- |
|  | | Frequency | Percent | Valid Percent | Cumulative Percent |
| Valid | <2 | 2 | 3.3 | 3.3 | 3.3 |
|  | 2-4.49 | 11 | 18.0 | 18.0 | 21.3 |
|  | 4.5-6.99 | 11 | 18.0 | 18.0 | 39.3 |
|  | 7-9.49 | 13 | 21.3 | 21.3 | 60.7 |
|  | >9.5 | 24 | 39.3 | 39.3 | 100.0 |
|  | Total | 61 | 100.0 | 100.0 |  |

FREQUENCIES VARIABLES=sex

/PIECHART FREQ

/ORDER=ANALYSIS.

**Frequencies**

| **Notes** | | |
| --- | --- | --- |
| Output Created | | 29-DEC-2022 14:08:50 |
| Comments | |  |
| Input | Data | C:\Users\user\Documents\asmare research_1.sav |
|  | Active Dataset | DataSet1 |
|  | Filter | <none> |
|  | Weight | <none> |
|  | Split File | <none> |
|  | N of Rows in Working Data File | 61 |
| Missing Value Handling | Definition of Missing | User-defined missing values are treated as missing. |
|  | Cases Used | Statistics are based on all cases with valid data. |
| Syntax | | FREQUENCIES VARIABLES=sex  /PIECHART FREQ  /ORDER=ANALYSIS. |
| Resources | Processor Time | 00:00:00.14 |
|  | Elapsed Time | 00:00:00.16 |

| **Statistics** | | |
| --- | --- | --- |
| Sex of child | | |
| N | Valid | 61 |
|  | Missing | 0 |

| **Sex of child** | | | | | |
| --- | --- | --- | --- | --- | --- |
|  | | Frequency | Percent | Valid Percent | Cumulative Percent |
| Valid | male | 24 | 39.3 | 39.3 | 39.3 |
|  | female | 37 | 60.7 | 60.7 | 100.0 |
|  | Total | 61 | 100.0 | 100.0 |  |


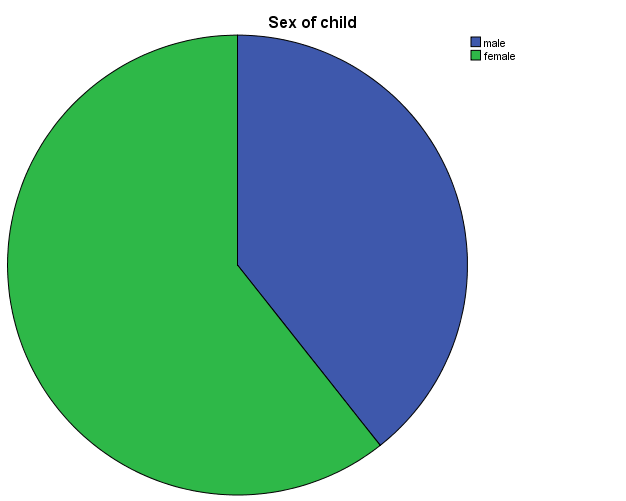


FREQUENCIES VARIABLES=educationalmothr martalstatus familyincome signesympyomdm withsymptomofdm parenetknowsignsymp firstdegrerelative infectionbeforedka signsymptomofinfection signsymptomofDKA newlydiagnosedtype1dm

/ORDER=ANALYSIS.

**Frequencies**

| **Notes** | | |
| --- | --- | --- |
| Output Created | | 29-DEC-2022 14:10:22 |
| Comments | |  |
| Input | Data | C:\Users\user\Documents\asmare research_1.sav |
|  | Active Dataset | DataSet1 |
|  | Filter | <none> |
|  | Weight | <none> |
|  | Split File | <none> |
|  | N of Rows in Working Data File | 61 |
| Missing Value Handling | Definition of Missing | User-defined missing values are treated as missing. |
|  | Cases Used | Statistics are based on all cases with valid data. |
| Syntax | | FREQUENCIES VARIABLES=educationalmothr martalstatus familyincome signesympyomdm withsymptomofdm parenetknowsignsymp firstdegrerelative infectionbeforedka signsymptomofinfection signsymptomofDKA newlydiagnosedtype1dm  /ORDER=ANALYSIS. |
| Resources | Processor Time | 00:00:00.02 |
|  | Elapsed Time | 00:00:00.01 |

| **Statistics** | | | | | | |
| --- | --- | --- | --- | --- | --- | --- |
|  | | Educational level of mother | Marital status of parents | Average family incomeper month | The child have sign and symptom of DM 2 week before the onset of dka | Which symptom of dm child presented with |
| N | Valid | 61 | 61 | 61 | 61 | 52 |
|  | Missing | 0 | 0 | 0 | 0 | 9 |

| **Statistics** | | | | | | |
| --- | --- | --- | --- | --- | --- | --- |
|  | | The child parent know sign and symptom of DM/DKA | Child have frist degre relative with dm | Child have sign and symptom of infection two week before onset of dka | Symptom of infection child present with | Symptom DKA the child present with |
| N | Valid | 61 | 61 | 61 | 21 | 37 |
|  | Missing | 0 | 0 | 0 | 40 | 24 |

| **Statistics** | | |
| --- | --- | --- |
|  | | Newly diagnosed Type 1DM |
| N | Valid | 61 |
|  | Missing | 0 |

**Frequency Table**

| **Educational level of mother** | | | | | |
| --- | --- | --- | --- | --- | --- |
|  | | Frequency | Percent | Valid Percent | Cumulative Percent |
| Valid | unable to read and write | 2 | 3.3 | 3.3 | 3.3 |
|  | read and write | 19 | 31.1 | 31.1 | 34.4 |
|  | grade1-8 | 19 | 31.1 | 31.1 | 65.6 |
|  | gread 9-12 | 16 | 26.2 | 26.2 | 91.8 |
|  | above 12 | 5 | 8.2 | 8.2 | 100.0 |
|  | Total | 61 | 100.0 | 100.0 |  |

| **Marital status of parents** | | | | | |
| --- | --- | --- | --- | --- | --- |
|  | | Frequency | Percent | Valid Percent | Cumulative Percent |
| Valid | single | 5 | 8.2 | 8.2 | 8.2 |
|  | married | 53 | 86.9 | 86.9 | 95.1 |
|  | separated | 2 | 3.3 | 3.3 | 98.4 |
|  | widowed | 1 | 1.6 | 1.6 | 100.0 |
|  | Total | 61 | 100.0 | 100.0 |  |

| **Average family incomeper month** | | | | | |
| --- | --- | --- | --- | --- | --- |
|  | | Frequency | Percent | Valid Percent | Cumulative Percent |
| Valid | <1000 | 4 | 6.6 | 6.6 | 6.6 |
|  | 1001-2200 | 19 | 31.1 | 31.1 | 37.7 |
|  | 2201-3400 | 9 | 14.8 | 14.8 | 52.5 |
|  | 3401-3600 | 5 | 8.2 | 8.2 | 60.7 |
|  | >3600 | 24 | 39.3 | 39.3 | 100.0 |
|  | Total | 61 | 100.0 | 100.0 |  |

| **The child have sign and symptom of DM 2 week before the onset of dka** | | | | | |
| --- | --- | --- | --- | --- | --- |
|  | | Frequency | Percent | Valid Percent | Cumulative Percent |
| Valid | yes | 52 | 85.2 | 85.2 | 85.2 |
|  | no | 9 | 14.8 | 14.8 | 100.0 |
|  | Total | 61 | 100.0 | 100.0 |  |

| **Which symptom of dm child presented with** | | | | | |
| --- | --- | --- | --- | --- | --- |
|  | | Frequency | Percent | Valid Percent | Cumulative Percent |
| Valid | polydepsia | 16 | 26.2 | 30.8 | 30.8 |
|  | polyuria | 7 | 11.5 | 13.5 | 44.2 |
|  | weight loss | 13 | 21.3 | 25.0 | 69.2 |
|  | all | 16 | 26.2 | 30.8 | 100.0 |
|  | Total | 52 | 85.2 | 100.0 |  |
| Missing | System | 9 | 14.8 |  |  |
| Total | | 61 | 100.0 |  |  |

| **The child parent know sign and symptom of DM/DKA** | | | | | |
| --- | --- | --- | --- | --- | --- |
|  | | Frequency | Percent | Valid Percent | Cumulative Percent |
| Valid | yes | 33 | 54.1 | 54.1 | 54.1 |
|  | no | 28 | 45.9 | 45.9 | 100.0 |
|  | Total | 61 | 100.0 | 100.0 |  |

| **Child have frist degre relative with dm** | | | | | |
| --- | --- | --- | --- | --- | --- |
|  | | Frequency | Percent | Valid Percent | Cumulative Percent |
| Valid | yes | 20 | 32.8 | 32.8 | 32.8 |
|  | no | 41 | 67.2 | 67.2 | 100.0 |
|  | Total | 61 | 100.0 | 100.0 |  |

| **Child have sign and symptom of infection two week before onset of dka** | | | | | |
| --- | --- | --- | --- | --- | --- |
|  | | Frequency | Percent | Valid Percent | Cumulative Percent |
| Valid | yes | 26 | 42.6 | 42.6 | 42.6 |
|  | no | 35 | 57.4 | 57.4 | 100.0 |
|  | Total | 61 | 100.0 | 100.0 |  |

| **Symptom of infection child present with** | | | | | |
| --- | --- | --- | --- | --- | --- |
|  | | Frequency | Percent | Valid Percent | Cumulative Percent |
| Valid | pnemonia | 7 | 11.5 | 33.3 | 33.3 |
|  | UTI | 4 | 6.6 | 19.0 | 52.4 |
|  | diarrhea | 6 | 9.8 | 28.6 | 81.0 |
|  | Acute tonsilopharengitis | 3 | 4.9 | 14.3 | 95.2 |
|  | other | 1 | 1.6 | 4.8 | 100.0 |
|  | Total | 21 | 34.4 | 100.0 |  |
| Missing | System | 40 | 65.6 |  |  |
| Total | | 61 | 100.0 |  |  |

| **Symptom DKA the child present with** | | | | | |
| --- | --- | --- | --- | --- | --- |
|  | | Frequency | Percent | Valid Percent | Cumulative Percent |
| Valid | abdominal pain | 10 | 16.4 | 27.0 | 27.0 |
|  | dehydration | 12 | 19.7 | 32.4 | 59.5 |
|  | loss of consciousness | 4 | 6.6 | 10.8 | 70.3 |
|  | vomiting | 2 | 3.3 | 5.4 | 75.7 |
|  | fast breathing | 9 | 14.8 | 24.3 | 100.0 |
|  | Total | 37 | 60.7 | 100.0 |  |
| Missing | System | 24 | 39.3 |  |  |
| Total | | 61 | 100.0 |  |  |
