# Supplementary material for: Pediatric Diabetic Ketoacidosis (PDKA) among newly diagnosed diabetic patients at Dilla University Hospital, Dilla, Ethiopia: Prevalence and predictors
Source: PLoS One. 2025 Jan 30;20(1):e0314433. doi: 10.1371/journal.pone.0314433 (PMC11781625; doi:10.1371/journal.pone.0314433)
Supplement: S2 File — (DOCX) [file pone.0314433.s002.docx]

| **Newly diagnosed Type 1DM** | | | | | |
| --- | --- | --- | --- | --- | --- |
|  | | Frequency | Percent | Valid Percent | Cumulative Percent |
| Valid | With DKA | 37 | 60.7 | 60.7 | 60.7 |
|  | Without DKA | 24 | 39.3 | 39.3 | 100.0 |
|  | Total | 61 | 100.0 | 100.0 |  |

LOGISTIC REGRESSION VARIABLES newlydiagnosedtype1dm

/METHOD=ENTER age

/CONTRAST (age)=Indicator(1)

/PRINT=GOODFIT CI(95)

/CRITERIA=PIN(0.05) POUT(0.10) ITERATE(20) CUT(0.5).

**Logistic Regression**

| **Notes** | | |
| --- | --- | --- |
| Output Created | | 29-DEC-2022 14:11:43 |
| Comments | |  |
| Input | Data | C:\Users\user\Documents\asmare research_1.sav |
|  | Active Dataset | DataSet1 |
|  | Filter | <none> |
|  | Weight | <none> |
|  | Split File | <none> |
|  | N of Rows in Working Data File | 61 |
| Missing Value Handling | Definition of Missing | User-defined missing values are treated as missing |
| Syntax | | LOGISTIC REGRESSION VARIABLES newlydiagnosedtype1dm  /METHOD=ENTER age  /CONTRAST (age)=Indicator(1)  /PRINT=GOODFIT CI(95)  /CRITERIA=PIN(0.05) POUT(0.10) ITERATE(20) CUT(0.5). |
| Resources | Processor Time | 00:00:00.02 |
|  | Elapsed Time | 00:00:00.02 |

| **Case Processing Summary** | | | | | | | | | | |  |  |
| --- | --- | --- | --- | --- | --- | --- | --- | --- | --- | --- | --- | --- |
| Unweighted Cases^a^ | | | | | | | N | | Percent | |  |  |
| Selected Cases | | | Included in Analysis | | | | 61 | | 100.0 | |  |  |
|  |  |  | Missing Cases | | | | 0 | | .0 | |  |  |
|  |  |  | Total | | | | 61 | | 100.0 | |  |  |
| Unselected Cases | | | | | | | 0 | | .0 | |  |  |
| Total | | | | | | | 61 | | 100.0 | |  |  |
| **Dependent Variable Encoding** | | | | |  |  |  |  |  |  |  |  |
| Original Value | | Internal Value | | |  |  |  |  |  |  |  |  |
| With DKA | | 0 | | |  |  |  |  |  |  |  |  |
| Without DKA | | 1 | | |  |  |  |  |  |  |  |  |
| **Categorical Variables Codings** | | | | | | | | | | | | |
|  | | | | Frequency | | Parameter coding | | | | | | |
|  |  |  |  |  |  | (1) | | (2) | | (3) | | (4) |
| Age of child | <2 | | | 2 | | .000 | | .000 | | .000 | | .000 |
|  | 2-4.49 | | | 11 | | 1.000 | | .000 | | .000 | | .000 |
|  | 4.5-6.99 | | | 11 | | .000 | | 1.000 | | .000 | | .000 |
|  | 7-9.49 | | | 13 | | .000 | | .000 | | 1.000 | | .000 |
|  | >9.5 | | | 24 | | .000 | | .000 | | .000 | | 1.000 |

**Block 0: Beginning Block**

| **Classification Table^a,b^** | | | | | | | | | | | | | | | | | |
| --- | --- | --- | --- | --- | --- | --- | --- | --- | --- | --- | --- | --- | --- | --- | --- | --- | --- |
|  | Observed | | | | | | | | Predicted | | | | | | | | |
|  |  |  |  |  |  |  |  |  | Newly diagnosed Type 1DM | | | | | | | Percentage Correct | |
|  |  |  |  |  |  |  |  |  | With DKA | | | | Without DKA | | |  |  |
| Step 0 | Newly diagnosed Type 1DM | | | | | With DKA | | | 37 | | | | 0 | | | 100.0 | |
|  |  |  |  |  |  | Without DKA | | | 24 | | | | 0 | | | .0 | |
|  | Overall Percentage | | | | | | | |  | | | |  | | | 60.7 | |
| a. Constant is included in the model. | | | | | | | | | | | | | | | | | |
| b. The cut value is .500 | | | | | | | | | | | | | | | | | |
| **Variables in the Equation** | | | | | | | | | | | | | | | | |  |
|  | | B | | | S.E. | | | Wald | | | df | | | Sig. | Exp(B) | |  |
| Step 0 | Constant | -.433 | | | .262 | | | 2.728 | | | 1 | | | .099 | .649 | |  |
| **Variables not in the Equation** | | | | | | | | | | | |  |  |  |  |  |  |
|  | | | | Score | | | df | | | Sig. | |  |  |  |  |  |  |
| Step 0 | Variables | | age | 1.226 | | | 4 | | | .874 | |  |  |  |  |  |  |
|  |  |  | age(1) | .819 | | | 1 | | | .365 | |  |  |  |  |  |  |
|  |  |  | age(2) | .210 | | | 1 | | | .647 | |  |  |  |  |  |  |
|  |  |  | age(3) | .321 | | | 1 | | | .571 | |  |  |  |  |  |  |
|  |  |  | age(4) | .056 | | | 1 | | | .812 | |  |  |  |  |  |  |
|  | Overall Statistics | | | 1.226 | | | 4 | | | .874 | |  |  |  |  |  |  |

**Block 1: Method = Enter**

| **Omnibus Tests of Model Coefficients** | | | | | | | |  |
| --- | --- | --- | --- | --- | --- | --- | --- | --- |
|  | | | Chi-square | | df | | Sig. |  |
| Step 1 | | Step | 1.250 | | 4 | | .870 |  |
|  |  | Block | 1.250 | | 4 | | .870 |  |
|  |  | Model | 1.250 | | 4 | | .870 |  |
| **Model Summary** | | | | | | | | |
| Step | -2 Log likelihood | | | Cox & Snell R Square | | Nagelkerke R Square | | |
| 1 | 80.522^a^ | | | .020 | | .027 | | |

a. Estimation terminated at iteration number 4 because parameter estimates changed by less than .001.

| **Hosmer and Lemeshow Test** | | | | | | | |  |  |  |  |  |
| --- | --- | --- | --- | --- | --- | --- | --- | --- | --- | --- | --- | --- |
| Step | Chi-square | | | df | | Sig. | |  |  |  |  |  |
| 1 | .000 | | | 2 | | 1.000 | |  |  |  |  |  |
| **Variables in the Equation** | | | | | | | | | | | | |
|  | | | B | | S.E. | | Wald | | df | Sig. | Exp(B) | 95% C.I.for EXP(B) |
|  |  |  |  |  |  |  |  |  |  |  |  | Lower |
| Step 1^a^ | | age |  | |  | | 1.203 | | 4 | .878 |  |  |
|  |  | age(1) | -.981 | | 1.568 | | .391 | | 1 | .532 | .375 | .017 |
|  |  | age(2) | -.182 | | 1.538 | | .014 | | 1 | .906 | .833 | .041 |
|  |  | age(3) | -.154 | | 1.520 | | .010 | | 1 | .919 | .857 | .044 |
|  |  | age(4) | -.511 | | 1.476 | | .120 | | 1 | .729 | .600 | .033 |
|  |  | Constant | .000 | | 1.414 | | .000 | | 1 | 1.000 | 1.000 |  |

a. Variable(s) entered on step 1: age.

LOGISTIC REGRESSION VARIABLES newlydiagnosedtype1dm

/METHOD=ENTER sex

/CONTRAST (sex)=Indicator

/PRINT=GOODFIT CI(95)

/CRITERIA=PIN(0.05) POUT(0.10) ITERATE(20) CUT(0.5).

**Logistic Regression**

| **Notes** | | |
| --- | --- | --- |
| Output Created | | 29-DEC-2022 14:12:42 |
| Comments | |  |
| Input | Data | C:\Users\user\Documents\asmare research_1.sav |
|  | Active Dataset | DataSet1 |
|  | Filter | <none> |
|  | Weight | <none> |
|  | Split File | <none> |
|  | N of Rows in Working Data File | 61 |
| Missing Value Handling | Definition of Missing | User-defined missing values are treated as missing |
| Syntax | | LOGISTIC REGRESSION VARIABLES newlydiagnosedtype1dm  /METHOD=ENTER sex  /CONTRAST (sex)=Indicator  /PRINT=GOODFIT CI(95)  /CRITERIA=PIN(0.05) POUT(0.10) ITERATE(20) CUT(0.5). |
| Resources | Processor Time | 00:00:00.00 |
|  | Elapsed Time | 00:00:00.00 |

| **Case Processing Summary** | | | |
| --- | --- | --- | --- |
| Unweighted Cases^a^ | | N | Percent |
| Selected Cases | Included in Analysis | 61 | 100.0 |
|  | Missing Cases | 0 | .0 |
|  | Total | 61 | 100.0 |
| Unselected Cases | | 0 | .0 |
| Total | | 61 | 100.0 |

a. If weight is in effect, see classification table for the total number of cases.

| **Dependent Variable Encoding** | | | |  |  |
| --- | --- | --- | --- | --- | --- |
| Original Value | | Internal Value | |  |  |
| With DKA | | 0 | |  |  |
| Without DKA | | 1 | |  |  |
| **Categorical Variables Codings** | | | | | |
|  | | | Frequency | | Parameter coding |
|  |  |  |  |  | (1) |
| Sex of child | male | | 24 | | 1.000 |
|  | female | | 37 | | .000 |

**Block 0: Beginning Block**

| **Classification Table^a,b^** | | | | | |
| --- | --- | --- | --- | --- | --- |
|  | Observed | | Predicted | | |
|  |  |  | Newly diagnosed Type 1DM | | Percentage Correct |
|  |  |  | With DKA | Without DKA |  |
| Step 0 | Newly diagnosed Type 1DM | With DKA | 37 | 0 | 100.0 |
|  |  | Without DKA | 24 | 0 | .0 |
|  | Overall Percentage | |  |  | 60.7 |
| a. Constant is included in the model. | | | | | |
| b. The cut value is .500 | | | | | |

**Block 1: Method = Enter**

| **Omnibus Tests of Model Coefficients** | | | | | | | |  |
| --- | --- | --- | --- | --- | --- | --- | --- | --- |
|  | | | Chi-square | | df | | Sig. |  |
| Step 1 | | Step | .695 | | 1 | | .404 |  |
|  |  | Block | .695 | | 1 | | .404 |  |
|  |  | Model | .695 | | 1 | | .404 |  |
| **Model Summary** | | | | | | | | |
| Step | -2 Log likelihood | | | Cox & Snell R Square | | Nagelkerke R Square | | |
| 1 | 81.077^a^ | | | .011 | | .015 | | |

a. Estimation terminated at iteration number 3 because parameter estimates changed by less than .001.

| **Hosmer and Lemeshow Test** | | | | | | | | |  |  |  |  |  |  |  |
| --- | --- | --- | --- | --- | --- | --- | --- | --- | --- | --- | --- | --- | --- | --- | --- |
| Step | Chi-square | | | df | | | Sig. | |  |  |  |  |  |  |  |
| 1 | .000 | | | 0 | | | . | |  |  |  |  |  |  |  |
| **Variables in the Equation** | | | | | | | | | | | | | | |  |
|  | | | B | | | S.E. | | Wald | | df | Sig. | | Exp(B) | 95% C.I.for EXP(B) |  |
|  |  |  |  |  |  |  |  |  |  |  |  |  |  | Lower |  |
| Step 1^a^ | | sex(1) | .446 | | | .535 | | .695 | | 1 | .405 | | 1.562 | .547 |  |
|  |  | Constant | -.613 | | | .344 | | 3.170 | | 1 | .075 | | .542 |  |  |
| **Variables in the Equation** | | | | | | | | | | | | | | | |
|  | | | | | | | | | | | | 95% C.I.for EXP(B) | | | |
|  |  |  |  |  |  |  |  |  |  |  |  | Upper | | | |
| Step 1^a^ | | | | | sex(1) | | | | | | | 4.459 | | | |
|  |  |  |  |  | Constant | | | | | | |  | | | |

LOGISTIC REGRESSION VARIABLES newlydiagnosedtype1dm

/METHOD=ENTER educationalmothr

/CONTRAST (educationalmothr)=Indicator

/PRINT=GOODFIT CI(95)

/CRITERIA=PIN(0.05) POUT(0.10) ITERATE(20) CUT(0.5).

**Logistic Regression**

| **Notes** | | |
| --- | --- | --- |
| Output Created | | 29-DEC-2022 14:16:57 |
| Comments | |  |
| Input | Data | C:\Users\user\Documents\asmare research_1.sav |
|  | Active Dataset | DataSet1 |
|  | Filter | <none> |
|  | Weight | <none> |
|  | Split File | <none> |
|  | N of Rows in Working Data File | 61 |
| Missing Value Handling | Definition of Missing | User-defined missing values are treated as missing |
| Syntax | | LOGISTIC REGRESSION VARIABLES newlydiagnosedtype1dm  /METHOD=ENTER educationalmothr  /CONTRAST (educationalmothr)=Indicator  /PRINT=GOODFIT CI(95)  /CRITERIA=PIN(0.05) POUT(0.10) ITERATE(20) CUT(0.5). |
| Resources | Processor Time | 00:00:00.02 |
|  | Elapsed Time | 00:00:00.02 |

| **Case Processing Summary** | | | |
| --- | --- | --- | --- |
| Unweighted Cases^a^ | | N | Percent |
| Selected Cases | Included in Analysis | 61 | 100.0 |
|  | Missing Cases | 0 | .0 |
|  | Total | 61 | 100.0 |
| Unselected Cases | | 0 | .0 |
| Total | | 61 | 100.0 |

| a. If weight is in effect, see classification table for the total number of cases. |
| --- |

| **Dependent Variable Encoding** | |
| --- | --- |
| Original Value | Internal Value |
| With DKA | 0 |
| Without DKA | 1 |

| **Categorical Variables Codings** | | | | | |
| --- | --- | --- | --- | --- | --- |
|  | | Frequency | Parameter coding | | |
|  |  |  | (1) | (2) | (3) |
| Educational level of mother | unable to read and write | 2 | 1.000 | .000 | .000 |
|  | read and write | 19 | .000 | 1.000 | .000 |
|  | grade1-8 | 19 | .000 | .000 | 1.000 |
|  | gread 9-12 | 16 | .000 | .000 | .000 |
|  | above 12 | 5 | .000 | .000 | .000 |

| **Categorical Variables Codings** | | |
| --- | --- | --- |
|  | | Parameter coding |
|  |  | (4) |
| Educational level of mother | unable to read and write | .000 |
|  | read and write | .000 |
|  | grade1-8 | .000 |
|  | gread 9-12 | 1.000 |
|  | above 12 | .000 |

**Block 0: Beginning Block**

| **Classification Table^a,b^** | | | | | |
| --- | --- | --- | --- | --- | --- |
|  | Observed | | Predicted | | |
|  |  |  | Newly diagnosed Type 1DM | | Percentage Correct |
|  |  |  | With DKA | Without DKA |  |
| Step 0 | Newly diagnosed Type 1DM | With DKA | 37 | 0 | 100.0 |
|  |  | Without DKA | 24 | 0 | .0 |
|  | Overall Percentage | |  |  | 60.7 |

| a. Constant is included in the model. |
| --- |
| b. The cut value is .500 |

| **Variables in the Equation** | | | | | | | |
| --- | --- | --- | --- | --- | --- | --- | --- |
|  | | B | S.E. | Wald | df | Sig. | Exp(B) |
| Step 0 | Constant | -.433 | .262 | 2.728 | 1 | .099 | .649 |

| **Variables not in the Equation** | | | | | |
| --- | --- | --- | --- | --- | --- |
|  | | | Score | df | Sig. |
| Step 0 | Variables | educationalmothr | 3.152 | 4 | .533 |
|  |  | educationalmothr(1) | .098 | 1 | .754 |
|  |  | educationalmothr(2) | .072 | 1 | .788 |
|  |  | educationalmothr(3) | 1.963 | 1 | .161 |
|  |  | educationalmothr(4) | 1.032 | 1 | .310 |
|  | Overall Statistics | | 3.152 | 4 | .533 |

**Block 1: Method = Enter**

| **Omnibus Tests of Model Coefficients** | | | | |
| --- | --- | --- | --- | --- |
|  | | Chi-square | df | Sig. |
| Step 1 | Step | 3.180 | 4 | .528 |
|  | Block | 3.180 | 4 | .528 |
|  | Model | 3.180 | 4 | .528 |

| **Model Summary** | | | |
| --- | --- | --- | --- |
| Step | -2 Log likelihood | Cox & Snell R Square | Nagelkerke R Square |
| 1 | 78.592^a^ | .051 | .069 |

| a. Estimation terminated at iteration number 4 because parameter estimates changed by less than .001. |
| --- |

| **Hosmer and Lemeshow Test** | | | |
| --- | --- | --- | --- |
| Step | Chi-square | df | Sig. |
| 1 | .000 | 2 | 1.000 |

| **Contingency Table for Hosmer and Lemeshow Test** | | | | | | |
| --- | --- | --- | --- | --- | --- | --- |
|  | | Newly diagnosed Type 1DM = With DKA | | Newly diagnosed Type 1DM = Without DKA | | Total |
|  |  | Observed | Expected | Observed | Expected |  |
| Step 1 | 1 | 14 | 14.000 | 5 | 5.000 | 19 |
|  | 2 | 12 | 12.000 | 7 | 7.000 | 19 |
|  | 3 | 9 | 9.000 | 9 | 9.000 | 18 |
|  | 4 | 2 | 2.000 | 3 | 3.000 | 5 |

| **Classification Table^a^** | | | | | |
| --- | --- | --- | --- | --- | --- |
|  | Observed | | Predicted | | |
|  |  |  | Newly diagnosed Type 1DM | | Percentage Correct |
|  |  |  | With DKA | Without DKA |  |
| Step 1 | Newly diagnosed Type 1DM | With DKA | 26 | 11 | 70.3 |
|  |  | Without DKA | 12 | 12 | 50.0 |
|  | Overall Percentage | |  |  | 62.3 |

| a. The cut value is .500 |
| --- |

| **Variables in the Equation** | | | | | | | |
| --- | --- | --- | --- | --- | --- | --- | --- |
|  | | B | S.E. | Wald | df | Sig. | Exp(B) |
|  |  |  |  |  |  |  |  |
| Step 1^a^ | educationalmothr |  |  | 3.042 | 4 | .551 |  |
|  | educationalmothr(1) | -.405 | 1.683 | .058 | 1 | .810 | .667 |
|  | educationalmothr(2) | -.944 | 1.029 | .842 | 1 | .359 | .389 |
|  | educationalmothr(3) | -1.435 | 1.051 | 1.864 | 1 | .172 | .238 |
|  | educationalmothr(4) | -.405 | 1.041 | .152 | 1 | .697 | .667 |
|  | Constant | .405 | .913 | .197 | 1 | .657 | 1.500 |

| **Variables in the Equation** | | | |
| --- | --- | --- | --- |
|  | | 95% C.I.for EXP(B) | |
|  |  | Lower | Upper |
| Step 1^a^ | educationalmothr |  |  |
|  | educationalmothr(1) | .025 | 18.059 |
|  | educationalmothr(2) | .052 | 2.924 |
|  | educationalmothr(3) | .030 | 1.868 |
|  | educationalmothr(4) | .087 | 5.127 |
|  | Constant |  |  |

| a. Variable(s) entered on step 1: educationalmothr. |
| --- |

LOGISTIC REGRESSION VARIABLES newlydiagnosedtype1dm

/METHOD=ENTER familyincome

/PRINT=GOODFIT CI(95)

/CRITERIA=PIN(0.05) POUT(0.10) ITERATE(20) CUT(0.5).

**Logistic Regression**

| **Notes** | | |
| --- | --- | --- |
| Output Created | | 29-DEC-2022 14:19:34 |
| Comments | |  |
| Input | Data | C:\Users\user\Documents\asmare research_1.sav |
|  | Active Dataset | DataSet1 |
|  | Filter | <none> |
|  | Weight | <none> |
|  | Split File | <none> |
|  | N of Rows in Working Data File | 61 |
| Missing Value Handling | Definition of Missing | User-defined missing values are treated as missing |
| Syntax | | LOGISTIC REGRESSION VARIABLES newlydiagnosedtype1dm  /METHOD=ENTER familyincome  /PRINT=GOODFIT CI(95)  /CRITERIA=PIN(0.05) POUT(0.10) ITERATE(20) CUT(0.5). |
| Resources | Processor Time | 00:00:00.00 |
|  | Elapsed Time | 00:00:00.00 |

| **Case Processing Summary** | | | |
| --- | --- | --- | --- |
| Unweighted Cases^a^ | | N | Percent |
| Selected Cases | Included in Analysis | 61 | 100.0 |
|  | Missing Cases | 0 | .0 |
|  | Total | 61 | 100.0 |
| Unselected Cases | | 0 | .0 |
| Total | | 61 | 100.0 |

| a. If weight is in effect, see classification table for the total number of cases. |
| --- |

| **Dependent Variable Encoding** | |
| --- | --- |
| Original Value | Internal Value |
| With DKA | 0 |
| Without DKA | 1 |

**Block 0: Beginning Block**

| **Classification Table^a,b^** | | | | | |
| --- | --- | --- | --- | --- | --- |
|  | Observed | | Predicted | | |
|  |  |  | Newly diagnosed Type 1DM | | Percentage Correct |
|  |  |  | With DKA | Without DKA |  |
| Step 0 | Newly diagnosed Type 1DM | With DKA | 37 | 0 | 100.0 |
|  |  | Without DKA | 24 | 0 | .0 |
|  | Overall Percentage | |  |  | 60.7 |

| a. Constant is included in the model. |
| --- |
| b. The cut value is .500 |

| **Variables in the Equation** | | | | | | | |
| --- | --- | --- | --- | --- | --- | --- | --- |
|  | | B | S.E. | Wald | df | Sig. | Exp(B) |
| Step 0 | Constant | -.433 | .262 | 2.728 | 1 | .099 | .649 |

| **Variables not in the Equation** | | | | | |
| --- | --- | --- | --- | --- | --- |
|  | | | Score | df | Sig. |
| Step 0 | Variables | familyincome | 14.472 | 1 | .000 |
|  | Overall Statistics | | 14.472 | 1 | .000 |

**Block 1: Method = Enter**

| **Omnibus Tests of Model Coefficients** | | | | |
| --- | --- | --- | --- | --- |
|  | | Chi-square | df | Sig. |
| Step 1 | Step | 15.638 | 1 | .000 |
|  | Block | 15.638 | 1 | .000 |
|  | Model | 15.638 | 1 | .000 |

| **Model Summary** | | | |
| --- | --- | --- | --- |
| Step | -2 Log likelihood | Cox & Snell R Square | Nagelkerke R Square |
| 1 | 66.134^a^ | .226 | .306 |

| a. Estimation terminated at iteration number 4 because parameter estimates changed by less than .001. |
| --- |

| **Hosmer and Lemeshow Test** | | | |
| --- | --- | --- | --- |
| Step | Chi-square | df | Sig. |
| 1 | 2.699 | 3 | .440 |

| **Contingency Table for Hosmer and Lemeshow Test** | | | | | | |
| --- | --- | --- | --- | --- | --- | --- |
|  | | Newly diagnosed Type 1DM = With DKA | | Newly diagnosed Type 1DM = Without DKA | | Total |
|  |  | Observed | Expected | Observed | Expected |  |
| Step 1 | 1 | 3 | 3.715 | 1 | .285 | 4 |
|  | 2 | 17 | 16.189 | 2 | 2.811 | 19 |
|  | 3 | 7 | 6.463 | 2 | 2.537 | 9 |
|  | 4 | 2 | 2.649 | 3 | 2.351 | 5 |
|  | 5 | 8 | 7.984 | 16 | 16.016 | 24 |

| **Classification Table^a^** | | | | | |
| --- | --- | --- | --- | --- | --- |
|  | Observed | | Predicted | | |
|  |  |  | Newly diagnosed Type 1DM | | Percentage Correct |
|  |  |  | With DKA | Without DKA |  |
| Step 1 | Newly diagnosed Type 1DM | With DKA | 29 | 8 | 78.4 |
|  |  | Without DKA | 8 | 16 | 66.7 |
|  | Overall Percentage | |  |  | 73.8 |

| a. The cut value is .500 |
| --- |

| **Variables in the Equation** | | | | | | | | |
| --- | --- | --- | --- | --- | --- | --- | --- | --- |
|  | | B | S.E. | Wald | df | Sig. | Exp(B) | 95% C.I.for EXP(B) |
|  |  |  |  |  |  |  |  | Lower |
| Step 1^a^ | familyincome | .816 | .231 | 12.456 | 1 | .000 | 2.261 | 1.437 |
|  | Constant | -3.382 | .931 | 13.200 | 1 | .000 | .034 |  |

| **Variables in the Equation** | | |
| --- | --- | --- |
|  | | 95% C.I.for EXP(B) |
|  |  | Upper |
| Step 1^a^ | familyincome | 3.556 |
|  | Constant |  |

| a. Variable(s) entered on step 1: familyincome. |
| --- |

LOGISTIC REGRESSION VARIABLES newlydiagnosedtype1dm

/METHOD=ENTER signesympyomdm

/CONTRAST (signesympyomdm)=Indicator(1)

/PRINT=GOODFIT CI(95)

/CRITERIA=PIN(0.05) POUT(0.10) ITERATE(20) CUT(0.5).

**Logistic Regression**

| **Notes** | | |
| --- | --- | --- |
| Output Created | | 29-DEC-2022 14:20:56 |
| Comments | |  |
| Input | Data | C:\Users\user\Documents\asmare research_1.sav |
|  | Active Dataset | DataSet1 |
|  | Filter | <none> |
|  | Weight | <none> |
|  | Split File | <none> |
|  | N of Rows in Working Data File | 61 |
| Missing Value Handling | Definition of Missing | User-defined missing values are treated as missing |
| Syntax | | LOGISTIC REGRESSION VARIABLES newlydiagnosedtype1dm  /METHOD=ENTER signesympyomdm  /CONTRAST (signesympyomdm)=Indicator(1)  /PRINT=GOODFIT CI(95)  /CRITERIA=PIN(0.05) POUT(0.10) ITERATE(20) CUT(0.5). |
| Resources | Processor Time | 00:00:00.00 |
|  | Elapsed Time | 00:00:00.00 |

| **Case Processing Summary** | | | |
| --- | --- | --- | --- |
| Unweighted Cases^a^ | | N | Percent |
| Selected Cases | Included in Analysis | 61 | 100.0 |
|  | Missing Cases | 0 | .0 |
|  | Total | 61 | 100.0 |
| Unselected Cases | | 0 | .0 |
| Total | | 61 | 100.0 |

| a. If weight is in effect, see classification table for the total number of cases. |
| --- |

| **Dependent Variable Encoding** | |
| --- | --- |
| Original Value | Internal Value |
| With DKA | 0 |
| Without DKA | 1 |

| **Categorical Variables Codings** | | | |
| --- | --- | --- | --- |
|  | | Frequency | Parameter coding |
|  |  |  | (1) |
| The child have sign and symptom of DM 2 week before the onset of dka | yes | 52 | .000 |
|  | no | 9 | 1.000 |

**Block 0: Beginning Block**

| **Classification Table^a,b^** | | | | | |
| --- | --- | --- | --- | --- | --- |
|  | Observed | | Predicted | | |
|  |  |  | Newly diagnosed Type 1DM | | Percentage Correct |
|  |  |  | With DKA | Without DKA |  |
| Step 0 | Newly diagnosed Type 1DM | With DKA | 37 | 0 | 100.0 |
|  |  | Without DKA | 24 | 0 | .0 |
|  | Overall Percentage | |  |  | 60.7 |

| a. Constant is included in the model. |
| --- |
| b. The cut value is .500 |

| **Variables in the Equation** | | | | | | | |
| --- | --- | --- | --- | --- | --- | --- | --- |
|  | | B | S.E. | Wald | df | Sig. | Exp(B) |
| Step 0 | Constant | -.433 | .262 | 2.728 | 1 | .099 | .649 |

| **Variables not in the Equation** | | | | | |
| --- | --- | --- | --- | --- | --- |
|  | | | Score | df | Sig. |
| Step 0 | Variables | signesympyomdm(1) | 3.526 | 1 | .060 |
|  | Overall Statistics | | 3.526 | 1 | .060 |

**Block 1: Method = Enter**

| **Omnibus Tests of Model Coefficients** | | | | |
| --- | --- | --- | --- | --- |
|  | | Chi-square | df | Sig. |
| Step 1 | Step | 4.100 | 1 | .043 |
|  | Block | 4.100 | 1 | .043 |
|  | Model | 4.100 | 1 | .043 |

| **Model Summary** | | | |
| --- | --- | --- | --- |
| Step | -2 Log likelihood | Cox & Snell R Square | Nagelkerke R Square |
| 1 | 77.672^a^ | .065 | .088 |

| a. Estimation terminated at iteration number 5 because parameter estimates changed by less than .001. |
| --- |

| **Hosmer and Lemeshow Test** | | | |
| --- | --- | --- | --- |
| Step | Chi-square | df | Sig. |
| 1 | .000 | 0 | . |

| **Contingency Table for Hosmer and Lemeshow Test** | | | | | | |
| --- | --- | --- | --- | --- | --- | --- |
|  | | Newly diagnosed Type 1DM = With DKA | | Newly diagnosed Type 1DM = Without DKA | | Total |
|  |  | Observed | Expected | Observed | Expected |  |
| Step 1 | 1 | 8 | 8.000 | 1 | 1.000 | 9 |
|  | 2 | 29 | 29.000 | 23 | 23.000 | 52 |

| **Classification Table^a^** | | | | | |
| --- | --- | --- | --- | --- | --- |
|  | Observed | | Predicted | | |
|  |  |  | Newly diagnosed Type 1DM | | Percentage Correct |
|  |  |  | With DKA | Without DKA |  |
| Step 1 | Newly diagnosed Type 1DM | With DKA | 37 | 0 | 100.0 |
|  |  | Without DKA | 24 | 0 | .0 |
|  | Overall Percentage | |  |  | 60.7 |

| a. The cut value is .500 |
| --- |

| **Variables in the Equation** | | | | | | | |
| --- | --- | --- | --- | --- | --- | --- | --- |
|  | | B | S.E. | Wald | df | Sig. | Exp(B) |
|  |  |  |  |  |  |  |  |
| Step 1^a^ | signesympyomdm(1) | -1.848 | 1.097 | 2.838 | 1 | .092 | .158 |
|  | Constant | -.232 | .279 | .689 | 1 | .406 | .793 |

| **Variables in the Equation** | | | |
| --- | --- | --- | --- |
|  | | 95% C.I.for EXP(B) | |
|  |  | Lower | Upper |
| Step 1^a^ | signesympyomdm(1) | .018 | 1.353 |
|  | Constant |  |  |

| a. Variable(s) entered on step 1: signesympyomdm. |
| --- |

LOGISTIC REGRESSION VARIABLES newlydiagnosedtype1dm

/METHOD=ENTER withsymptomofdm

/PRINT=GOODFIT CI(95)

/CRITERIA=PIN(0.05) POUT(0.10) ITERATE(20) CUT(0.5).

**Logistic Regression**

| **Notes** | | |
| --- | --- | --- |
| Output Created | | 29-DEC-2022 14:21:57 |
| Comments | |  |
| Input | Data | C:\Users\user\Documents\asmare research_1.sav |
|  | Active Dataset | DataSet1 |
|  | Filter | <none> |
|  | Weight | <none> |
|  | Split File | <none> |
|  | N of Rows in Working Data File | 61 |
| Missing Value Handling | Definition of Missing | User-defined missing values are treated as missing |
| Syntax | | LOGISTIC REGRESSION VARIABLES newlydiagnosedtype1dm  /METHOD=ENTER withsymptomofdm  /PRINT=GOODFIT CI(95)  /CRITERIA=PIN(0.05) POUT(0.10) ITERATE(20) CUT(0.5). |
| Resources | Processor Time | 00:00:00.00 |
|  | Elapsed Time | 00:00:00.00 |

| **Case Processing Summary** | | | |
| --- | --- | --- | --- |
| Unweighted Cases^a^ | | N | Percent |
| Selected Cases | Included in Analysis | 52 | 85.2 |
|  | Missing Cases | 9 | 14.8 |
|  | Total | 61 | 100.0 |
| Unselected Cases | | 0 | .0 |
| Total | | 61 | 100.0 |

| a. If weight is in effect, see classification table for the total number of cases. |
| --- |

| **Dependent Variable Encoding** | |
| --- | --- |
| Original Value | Internal Value |
| With DKA | 0 |
| Without DKA | 1 |

**Block 0: Beginning Block**

| **Classification Table^a,b^** | | | | | |
| --- | --- | --- | --- | --- | --- |
|  | Observed | | Predicted | | |
|  |  |  | Newly diagnosed Type 1DM | | Percentage Correct |
|  |  |  | With DKA | Without DKA |  |
| Step 0 | Newly diagnosed Type 1DM | With DKA | 29 | 0 | 100.0 |
|  |  | Without DKA | 23 | 0 | .0 |
|  | Overall Percentage | |  |  | 55.8 |

| a. Constant is included in the model. |
| --- |
| b. The cut value is .500 |

| **Variables in the Equation** | | | | | | | |
| --- | --- | --- | --- | --- | --- | --- | --- |
|  | | B | S.E. | Wald | df | Sig. | Exp(B) |
| Step 0 | Constant | -.232 | .279 | .689 | 1 | .406 | .793 |

| **Variables not in the Equation** | | | | | |
| --- | --- | --- | --- | --- | --- |
|  | | | Score | df | Sig. |
| Step 0 | Variables | withsymptomofdm | .176 | 1 | .675 |
|  | Overall Statistics | | .176 | 1 | .675 |

**Block 1: Method = Enter**

| **Omnibus Tests of Model Coefficients** | | | | |
| --- | --- | --- | --- | --- |
|  | | Chi-square | df | Sig. |
| Step 1 | Step | .176 | 1 | .675 |
|  | Block | .176 | 1 | .675 |
|  | Model | .176 | 1 | .675 |

| **Model Summary** | | | |
| --- | --- | --- | --- |
| Step | -2 Log likelihood | Cox & Snell R Square | Nagelkerke R Square |
| 1 | 71.217^a^ | .003 | .005 |

| a. Estimation terminated at iteration number 3 because parameter estimates changed by less than .001. |
| --- |

| **Hosmer and Lemeshow Test** | | | |
| --- | --- | --- | --- |
| Step | Chi-square | df | Sig. |
| 1 | .222 | 2 | .895 |

| **Contingency Table for Hosmer and Lemeshow Test** | | | | | | |
| --- | --- | --- | --- | --- | --- | --- |
|  | | Newly diagnosed Type 1DM = With DKA | | Newly diagnosed Type 1DM = Without DKA | | Total |
|  |  | Observed | Expected | Observed | Expected |  |
| Step 1 | 1 | 9 | 9.470 | 7 | 6.530 | 16 |
|  | 2 | 8 | 7.389 | 5 | 5.611 | 13 |
|  | 3 | 4 | 3.812 | 3 | 3.188 | 7 |
|  | 4 | 8 | 8.329 | 8 | 7.671 | 16 |

| **Classification Table^a^** | | | | | |
| --- | --- | --- | --- | --- | --- |
|  | Observed | | Predicted | | |
|  |  |  | Newly diagnosed Type 1DM | | Percentage Correct |
|  |  |  | With DKA | Without DKA |  |
| Step 1 | Newly diagnosed Type 1DM | With DKA | 29 | 0 | 100.0 |
|  |  | Without DKA | 23 | 0 | .0 |
|  | Overall Percentage | |  |  | 55.8 |

| a. The cut value is .500 |
| --- |

| **Variables in the Equation** | | | | | | | |
| --- | --- | --- | --- | --- | --- | --- | --- |
|  | | B | S.E. | Wald | df | Sig. | Exp(B) |
|  |  |  |  |  |  |  |  |
| Step 1^a^ | withsymptomofdm | -.096 | .230 | .176 | 1 | .675 | .908 |
|  | Constant | .014 | .648 | .000 | 1 | .983 | 1.014 |

| **Variables in the Equation** | | | |
| --- | --- | --- | --- |
|  | | 95% C.I.for EXP(B) | |
|  |  | Lower | Upper |
| Step 1^a^ | withsymptomofdm | .578 | 1.425 |
|  | Constant |  |  |

| a. Variable(s) entered on step 1: withsymptomofdm. |
| --- |

LOGISTIC REGRESSION VARIABLES newlydiagnosedtype1dm

/METHOD=ENTER firstdegrerelative

/CONTRAST (firstdegrerelative)=Indicator(1)

/PRINT=GOODFIT CI(95)

/CRITERIA=PIN(0.05) POUT(0.10) ITERATE(20) CUT(0.5).

**Logistic Regression**

| **Notes** | | |
| --- | --- | --- |
| Output Created | | 29-DEC-2022 14:23:05 |
| Comments | |  |
| Input | Data | C:\Users\user\Documents\asmare research_1.sav |
|  | Active Dataset | DataSet1 |
|  | Filter | <none> |
|  | Weight | <none> |
|  | Split File | <none> |
|  | N of Rows in Working Data File | 61 |
| Missing Value Handling | Definition of Missing | User-defined missing values are treated as missing |
| Syntax | | LOGISTIC REGRESSION VARIABLES newlydiagnosedtype1dm  /METHOD=ENTER firstdegrerelative  /CONTRAST (firstdegrerelative)=Indicator(1)  /PRINT=GOODFIT CI(95)  /CRITERIA=PIN(0.05) POUT(0.10) ITERATE(20) CUT(0.5). |
| Resources | Processor Time | 00:00:00.02 |
|  | Elapsed Time | 00:00:00.02 |

| **Case Processing Summary** | | | |
| --- | --- | --- | --- |
| Unweighted Cases^a^ | | N | Percent |
| Selected Cases | Included in Analysis | 61 | 100.0 |
|  | Missing Cases | 0 | .0 |
|  | Total | 61 | 100.0 |
| Unselected Cases | | 0 | .0 |
| Total | | 61 | 100.0 |

| a. If weight is in effect, see classification table for the total number of cases. |
| --- |

| **Dependent Variable Encoding** | |
| --- | --- |
| Original Value | Internal Value |
| With DKA | 0 |
| Without DKA | 1 |

| **Categorical Variables Codings** | | | |
| --- | --- | --- | --- |
|  | | Frequency | Parameter coding |
|  |  |  | (1) |
| Child have frist degre relative with dm | yes | 20 | .000 |
|  | no | 41 | 1.000 |

**Block 0: Beginning Block**

| **Classification Table^a,b^** | | | | | |
| --- | --- | --- | --- | --- | --- |
|  | Observed | | Predicted | | |
|  |  |  | Newly diagnosed Type 1DM | | Percentage Correct |
|  |  |  | With DKA | Without DKA |  |
| Step 0 | Newly diagnosed Type 1DM | With DKA | 37 | 0 | 100.0 |
|  |  | Without DKA | 24 | 0 | .0 |
|  | Overall Percentage | |  |  | 60.7 |

| a. Constant is included in the model. |
| --- |
| b. The cut value is .500 |

| **Variables in the Equation** | | | | | | | |
| --- | --- | --- | --- | --- | --- | --- | --- |
|  | | B | S.E. | Wald | df | Sig. | Exp(B) |
| Step 0 | Constant | -.433 | .262 | 2.728 | 1 | .099 | .649 |

| **Variables not in the Equation** | | | | | |
| --- | --- | --- | --- | --- | --- |
|  | | | Score | df | Sig. |
| Step 0 | Variables | firstdegrerelative(1) | 8.207 | 1 | .004 |
|  | Overall Statistics | | 8.207 | 1 | .004 |

**Block 1: Method = Enter**

| **Omnibus Tests of Model Coefficients** | | | | |
| --- | --- | --- | --- | --- |
|  | | Chi-square | df | Sig. |
| Step 1 | Step | 8.187 | 1 | .004 |
|  | Block | 8.187 | 1 | .004 |
|  | Model | 8.187 | 1 | .004 |

| **Model Summary** | | | |
| --- | --- | --- | --- |
| Step | -2 Log likelihood | Cox & Snell R Square | Nagelkerke R Square |
| 1 | 73.585^a^ | .126 | .170 |

| a. Estimation terminated at iteration number 4 because parameter estimates changed by less than .001. |
| --- |

| **Hosmer and Lemeshow Test** | | | |
| --- | --- | --- | --- |
| Step | Chi-square | df | Sig. |
| 1 | .000 | 0 | . |

| **Contingency Table for Hosmer and Lemeshow Test** | | | | | | |
| --- | --- | --- | --- | --- | --- | --- |
|  | | Newly diagnosed Type 1DM = With DKA | | Newly diagnosed Type 1DM = Without DKA | | Total |
|  |  | Observed | Expected | Observed | Expected |  |
| Step 1 | 1 | 30 | 30.000 | 11 | 11.000 | 41 |
|  | 2 | 7 | 7.000 | 13 | 13.000 | 20 |

| **Classification Table^a^** | | | | | |
| --- | --- | --- | --- | --- | --- |
|  | Observed | | Predicted | | |
|  |  |  | Newly diagnosed Type 1DM | | Percentage Correct |
|  |  |  | With DKA | Without DKA |  |
| Step 1 | Newly diagnosed Type 1DM | With DKA | 30 | 7 | 81.1 |
|  |  | Without DKA | 11 | 13 | 54.2 |
|  | Overall Percentage | |  |  | 70.5 |

| a. The cut value is .500 |
| --- |

| **Variables in the Equation** | | | | | | | |
| --- | --- | --- | --- | --- | --- | --- | --- |
|  | | B | S.E. | Wald | df | Sig. | Exp(B) |
|  |  |  |  |  |  |  |  |
| Step 1^a^ | firstdegrerelative(1) | -1.622 | .587 | 7.651 | 1 | .006 | .197 |
|  | Constant | .619 | .469 | 1.744 | 1 | .187 | 1.857 |

| **Variables in the Equation** | | | |
| --- | --- | --- | --- |
|  | | 95% C.I.for EXP(B) | |
|  |  | Lower | Upper |
| Step 1^a^ | firstdegrerelative(1) | .063 | .623 |
|  | Constant |  |  |

| a. Variable(s) entered on step 1: firstdegrerelative. |
| --- |

LOGISTIC REGRESSION VARIABLES newlydiagnosedtype1dm

/METHOD=ENTER parenetknowsignsymp

/CONTRAST (parenetknowsignsymp)=Indicator(1)

/PRINT=GOODFIT CI(95)

/CRITERIA=PIN(0.05) POUT(0.10) ITERATE(20) CUT(0.5).

**Logistic Regression**

| **Notes** | | |
| --- | --- | --- |
| Output Created | | 29-DEC-2022 14:23:44 |
| Comments | |  |
| Input | Data | C:\Users\user\Documents\asmare research_1.sav |
|  | Active Dataset | DataSet1 |
|  | Filter | <none> |
|  | Weight | <none> |
|  | Split File | <none> |
|  | N of Rows in Working Data File | 61 |
| Missing Value Handling | Definition of Missing | User-defined missing values are treated as missing |
| Syntax | | LOGISTIC REGRESSION VARIABLES newlydiagnosedtype1dm  /METHOD=ENTER parenetknowsignsymp  /CONTRAST (parenetknowsignsymp)=Indicator(1)  /PRINT=GOODFIT CI(95)  /CRITERIA=PIN(0.05) POUT(0.10) ITERATE(20) CUT(0.5). |
| Resources | Processor Time | 00:00:00.02 |
|  | Elapsed Time | 00:00:00.02 |

| **Case Processing Summary** | | | |
| --- | --- | --- | --- |
| Unweighted Cases^a^ | | N | Percent |
| Selected Cases | Included in Analysis | 61 | 100.0 |
|  | Missing Cases | 0 | .0 |
|  | Total | 61 | 100.0 |
| Unselected Cases | | 0 | .0 |
| Total | | 61 | 100.0 |

| a. If weight is in effect, see classification table for the total number of cases. |
| --- |

| **Dependent Variable Encoding** | |
| --- | --- |
| Original Value | Internal Value |
| With DKA | 0 |
| Without DKA | 1 |

| **Categorical Variables Codings** | | | |
| --- | --- | --- | --- |
|  | | Frequency | Parameter coding |
|  |  |  | (1) |
| The child parent know sign and symptom of DM/DKA | yes | 33 | .000 |
|  | no | 28 | 1.000 |

**Block 0: Beginning Block**

| **Classification Table^a,b^** | | | | | |
| --- | --- | --- | --- | --- | --- |
|  | Observed | | Predicted | | |
|  |  |  | Newly diagnosed Type 1DM | | Percentage Correct |
|  |  |  | With DKA | Without DKA |  |
| Step 0 | Newly diagnosed Type 1DM | With DKA | 37 | 0 | 100.0 |
|  |  | Without DKA | 24 | 0 | .0 |
|  | Overall Percentage | |  |  | 60.7 |

| a. Constant is included in the model. |
| --- |
| b. The cut value is .500 |

| **Variables in the Equation** | | | | | | | |
| --- | --- | --- | --- | --- | --- | --- | --- |
|  | | B | S.E. | Wald | df | Sig. | Exp(B) |
| Step 0 | Constant | -.433 | .262 | 2.728 | 1 | .099 | .649 |

| **Variables not in the Equation** | | | | | |
| --- | --- | --- | --- | --- | --- |
|  | | | Score | df | Sig. |
| Step 0 | Variables | parenetknowsignsymp(1) | 17.777 | 1 | .000 |
|  | Overall Statistics | | 17.777 | 1 | .000 |

**Block 1: Method = Enter**

| **Omnibus Tests of Model Coefficients** | | | | |
| --- | --- | --- | --- | --- |
|  | | Chi-square | df | Sig. |
| Step 1 | Step | 19.442 | 1 | .000 |
|  | Block | 19.442 | 1 | .000 |
|  | Model | 19.442 | 1 | .000 |

| **Model Summary** | | | |
| --- | --- | --- | --- |
| Step | -2 Log likelihood | Cox & Snell R Square | Nagelkerke R Square |
| 1 | 62.330^a^ | .273 | .370 |

| a. Estimation terminated at iteration number 5 because parameter estimates changed by less than .001. |
| --- |

| **Hosmer and Lemeshow Test** | | | |
| --- | --- | --- | --- |
| Step | Chi-square | df | Sig. |
| 1 | .000 | 0 | . |

| **Contingency Table for Hosmer and Lemeshow Test** | | | | | | |
| --- | --- | --- | --- | --- | --- | --- |
|  | | Newly diagnosed Type 1DM = With DKA | | Newly diagnosed Type 1DM = Without DKA | | Total |
|  |  | Observed | Expected | Observed | Expected |  |
| Step 1 | 1 | 25 | 25.000 | 3 | 3.000 | 28 |
|  | 2 | 12 | 12.000 | 21 | 21.000 | 33 |

| **Classification Table^a^** | | | | | |
| --- | --- | --- | --- | --- | --- |
|  | Observed | | Predicted | | |
|  |  |  | Newly diagnosed Type 1DM | | Percentage Correct |
|  |  |  | With DKA | Without DKA |  |
| Step 1 | Newly diagnosed Type 1DM | With DKA | 25 | 12 | 67.6 |
|  |  | Without DKA | 3 | 21 | 87.5 |
|  | Overall Percentage | |  |  | 75.4 |

| a. The cut value is .500 |
| --- |

| **Variables in the Equation** | | | | | | | |
| --- | --- | --- | --- | --- | --- | --- | --- |
|  | | B | S.E. | Wald | df | Sig. | Exp(B) |
|  |  |  |  |  |  |  |  |
| Step 1^a^ | parenetknowsignsymp(1) | -2.680 | .710 | 14.241 | 1 | .000 | .069 |
|  | Constant | .560 | .362 | 2.391 | 1 | .122 | 1.750 |

| **Variables in the Equation** | | | |
| --- | --- | --- | --- |
|  | | 95% C.I.for EXP(B) | |
|  |  | Lower | Upper |
| Step 1^a^ | parenetknowsignsymp(1) | .017 | .276 |
|  | Constant |  |  |

| a. Variable(s) entered on step 1: parenetknowsignsymp. |
| --- |

LOGISTIC REGRESSION VARIABLES newlydiagnosedtype1dm

/METHOD=ENTER infectionbeforedka

/CONTRAST (infectionbeforedka)=Indicator(1)

/PRINT=GOODFIT CI(95)

/CRITERIA=PIN(0.05) POUT(0.10) ITERATE(20) CUT(0.5).

**Logistic Regression**

| **Notes** | | |
| --- | --- | --- |
| Output Created | | 29-DEC-2022 14:24:51 |
| Comments | |  |
| Input | Data | C:\Users\user\Documents\asmare research_1.sav |
|  | Active Dataset | DataSet1 |
|  | Filter | <none> |
|  | Weight | <none> |
|  | Split File | <none> |
|  | N of Rows in Working Data File | 61 |
| Missing Value Handling | Definition of Missing | User-defined missing values are treated as missing |
| Syntax | | LOGISTIC REGRESSION VARIABLES newlydiagnosedtype1dm  /METHOD=ENTER infectionbeforedka  /CONTRAST (infectionbeforedka)=Indicator(1)  /PRINT=GOODFIT CI(95)  /CRITERIA=PIN(0.05) POUT(0.10) ITERATE(20) CUT(0.5). |
| Resources | Processor Time | 00:00:00.00 |
|  | Elapsed Time | 00:00:00.02 |

| **Case Processing Summary** | | | |
| --- | --- | --- | --- |
| Unweighted Cases^a^ | | N | Percent |
| Selected Cases | Included in Analysis | 61 | 100.0 |
|  | Missing Cases | 0 | .0 |
|  | Total | 61 | 100.0 |
| Unselected Cases | | 0 | .0 |
| Total | | 61 | 100.0 |

| a. If weight is in effect, see classification table for the total number of cases. |
| --- |

| **Dependent Variable Encoding** | |
| --- | --- |
| Original Value | Internal Value |
| With DKA | 0 |
| Without DKA | 1 |

| **Categorical Variables Codings** | | | |
| --- | --- | --- | --- |
|  | | Frequency | Parameter coding |
|  |  |  | (1) |
| Child have sign and symptom of infection two week before onset of dka | yes | 26 | .000 |
|  | no | 35 | 1.000 |

**Block 0: Beginning Block**

| **Classification Table^a,b^** | | | | | |
| --- | --- | --- | --- | --- | --- |
|  | Observed | | Predicted | | |
|  |  |  | Newly diagnosed Type 1DM | | Percentage Correct |
|  |  |  | With DKA | Without DKA |  |
| Step 0 | Newly diagnosed Type 1DM | With DKA | 37 | 0 | 100.0 |
|  |  | Without DKA | 24 | 0 | .0 |
|  | Overall Percentage | |  |  | 60.7 |

| a. Constant is included in the model. |
| --- |
| b. The cut value is .500 |

| **Variables in the Equation** | | | | | | | |
| --- | --- | --- | --- | --- | --- | --- | --- |
|  | | B | S.E. | Wald | df | Sig. | Exp(B) |
| Step 0 | Constant | -.433 | .262 | 2.728 | 1 | .099 | .649 |

| **Variables not in the Equation** | | | | | |
| --- | --- | --- | --- | --- | --- |
|  | | | Score | df | Sig. |
| Step 0 | Variables | infectionbeforedka(1) | 7.682 | 1 | .006 |
|  | Overall Statistics | | 7.682 | 1 | .006 |

**Block 1: Method = Enter**

| **Omnibus Tests of Model Coefficients** | | | | |
| --- | --- | --- | --- | --- |
|  | | Chi-square | df | Sig. |
| Step 1 | Step | 8.053 | 1 | .005 |
|  | Block | 8.053 | 1 | .005 |
|  | Model | 8.053 | 1 | .005 |

| **Model Summary** | | | |
| --- | --- | --- | --- |
| Step | -2 Log likelihood | Cox & Snell R Square | Nagelkerke R Square |
| 1 | 73.720^a^ | .124 | .168 |

| a. Estimation terminated at iteration number 4 because parameter estimates changed by less than .001. |
| --- |

| **Hosmer and Lemeshow Test** | | | |
| --- | --- | --- | --- |
| Step | Chi-square | df | Sig. |
| 1 | .000 | 0 | . |

| **Contingency Table for Hosmer and Lemeshow Test** | | | | | | |
| --- | --- | --- | --- | --- | --- | --- |
|  | | Newly diagnosed Type 1DM = With DKA | | Newly diagnosed Type 1DM = Without DKA | | Total |
|  |  | Observed | Expected | Observed | Expected |  |
| Step 1 | 1 | 21 | 21.000 | 5 | 5.000 | 26 |
|  | 2 | 16 | 16.000 | 19 | 19.000 | 35 |

| **Classification Table^a^** | | | | | |
| --- | --- | --- | --- | --- | --- |
|  | Observed | | Predicted | | |
|  |  |  | Newly diagnosed Type 1DM | | Percentage Correct |
|  |  |  | With DKA | Without DKA |  |
| Step 1 | Newly diagnosed Type 1DM | With DKA | 21 | 16 | 56.8 |
|  |  | Without DKA | 5 | 19 | 79.2 |
|  | Overall Percentage | |  |  | 65.6 |

| a. The cut value is .500 |
| --- |

| **Variables in the Equation** | | | | | | | |
| --- | --- | --- | --- | --- | --- | --- | --- |
|  | | B | S.E. | Wald | df | Sig. | Exp(B) |
|  |  |  |  |  |  |  |  |
| Step 1^a^ | infectionbeforedka(1) | 1.607 | .602 | 7.118 | 1 | .008 | 4.987 |
|  | Constant | -1.435 | .498 | 8.317 | 1 | .004 | .238 |

| **Variables in the Equation** | | | |
| --- | --- | --- | --- |
|  | | 95% C.I.for EXP(B) | |
|  |  | Lower | Upper |
| Step 1^a^ | infectionbeforedka(1) | 1.532 | 16.239 |
|  | Constant |  |  |

| a. Variable(s) entered on step 1: infectionbeforedka. |
| --- |

LOGISTIC REGRESSION VARIABLES newlydiagnosedtype1dm

/METHOD=ENTER infectionbeforedka educfather familyincome firstdegrerelative parenetknowsignsymp

/CONTRAST (infectionbeforedka)=Indicator(1)

/CONTRAST (educfather)=Indicator(1)

/CONTRAST (firstdegrerelative)=Indicator(1)

/CONTRAST (parenetknowsignsymp)=Indicator(1)

/PRINT=GOODFIT CI(95)

/CRITERIA=PIN(0.05) POUT(0.10) ITERATE(20) CUT(0.5).

**Logistic Regression**

| **Notes** | | |
| --- | --- | --- |
| Output Created | | 29-DEC-2022 14:35:19 |
| Comments | |  |
| Input | Data | C:\Users\user\Documents\asmare research_1.sav |
|  | Active Dataset | DataSet1 |
|  | Filter | <none> |
|  | Weight | <none> |
|  | Split File | <none> |
|  | N of Rows in Working Data File | 61 |
| Missing Value Handling | Definition of Missing | User-defined missing values are treated as missing |
| Syntax | | LOGISTIC REGRESSION VARIABLES newlydiagnosedtype1dm  /METHOD=ENTER infectionbeforedka educfather familyincome firstdegrerelative parenetknowsignsymp  /CONTRAST (infectionbeforedka)=Indicator(1)  /CONTRAST (educfather)=Indicator(1)  /CONTRAST (firstdegrerelative)=Indicator(1)  /CONTRAST (parenetknowsignsymp)=Indicator(1)  /PRINT=GOODFIT CI(95)  /CRITERIA=PIN(0.05) POUT(0.10) ITERATE(20) CUT(0.5). |
| Resources | Processor Time | 00:00:00.02 |
|  | Elapsed Time | 00:00:00.01 |

| **Case Processing Summary** | | | |
| --- | --- | --- | --- |
| Unweighted Cases^a^ | | N | Percent |
| Selected Cases | Included in Analysis | 61 | 100.0 |
|  | Missing Cases | 0 | .0 |
|  | Total | 61 | 100.0 |
| Unselected Cases | | 0 | .0 |
| Total | | 61 | 100.0 |

| a. If weight is in effect, see classification table for the total number of cases. |
| --- |

| **Dependent Variable Encoding** | |
| --- | --- |
| Original Value | Internal Value |
| With DKA | 0 |
| Without DKA | 1 |

| **Categorical Variables Codings** | | | | | |
| --- | --- | --- | --- | --- | --- |
|  | | Frequency | Parameter coding | | |
|  |  |  | (1) | (2) | (3) |
| Educational level of father | unable to read and write | 1 | .000 | .000 | .000 |
|  | read and write | 10 | 1.000 | .000 | .000 |
|  | gread1- 8 | 14 | .000 | 1.000 | .000 |
|  | gread 9-12 | 16 | .000 | .000 | 1.000 |
|  | above 12 | 20 | .000 | .000 | .000 |
| The child parent know sign and symptom of DM/DKA | yes | 33 | .000 |  |  |
|  | no | 28 | 1.000 |  |  |
| Child have frist degre relative with dm | yes | 20 | .000 |  |  |
|  | no | 41 | 1.000 |  |  |
| Child have sign and symptom of infection two week before onset of dka | yes | 26 | .000 |  |  |
|  | no | 35 | 1.000 |  |  |

| **Categorical Variables Codings** | | |
| --- | --- | --- |
|  | | Parameter coding |
|  |  | (4) |
| Educational level of father | unable to read and write | .000 |
|  | read and write | .000 |
|  | gread1- 8 | .000 |
|  | gread 9-12 | .000 |
|  | above 12 | 1.000 |
| The child parent know sign and symptom of DM/DKA | yes |  |
|  | no |  |
| Child have frist degre relative with dm | yes |  |
|  | no |  |
| Child have sign and symptom of infection two week before onset of dka | yes |  |
|  | no |  |

**Block 0: Beginning Block**

| **Classification Table^a,b^** | | | | | |
| --- | --- | --- | --- | --- | --- |
|  | Observed | | Predicted | | |
|  |  |  | Newly diagnosed Type 1DM | | Percentage Correct |
|  |  |  | With DKA | Without DKA |  |
| Step 0 | Newly diagnosed Type 1DM | With DKA | 37 | 0 | 100.0 |
|  |  | Without DKA | 24 | 0 | .0 |
|  | Overall Percentage | |  |  | 60.7 |

| a. Constant is included in the model. |
| --- |
| b. The cut value is .500 |

| **Variables in the Equation** | | | | | | | |
| --- | --- | --- | --- | --- | --- | --- | --- |
|  | | B | S.E. | Wald | df | Sig. | Exp(B) |
| Step 0 | Constant | -.433 | .262 | 2.728 | 1 | .099 | .649 |

| **Variables not in the Equation** | | | | | |
| --- | --- | --- | --- | --- | --- |
|  | | | Score | df | Sig. |
| Step 0 | Variables | infectionbeforedka(1) | 7.682 | 1 | .006 |
|  |  | educfather | 18.491 | 4 | .001 |
|  |  | educfather(1) | 1.875 | 1 | .171 |
|  |  | educfather(2) | 2.444 | 1 | .118 |
|  |  | educfather(3) | 3.855 | 1 | .050 |
|  |  | educfather(4) | 15.852 | 1 | .000 |
|  |  | familyincome | 14.472 | 1 | .000 |
|  |  | firstdegrerelative(1) | 8.207 | 1 | .004 |
|  |  | parenetknowsignsymp(1) | 17.777 | 1 | .000 |
|  | Overall Statistics | | 34.615 | 8 | .000 |

**Block 1: Method = Enter**

| **Omnibus Tests of Model Coefficients** | | | | |
| --- | --- | --- | --- | --- |
|  | | Chi-square | df | Sig. |
| Step 1 | Step | 46.952 | 8 | .000 |
|  | Block | 46.952 | 8 | .000 |
|  | Model | 46.952 | 8 | .000 |

| **Model Summary** | | | |
| --- | --- | --- | --- |
| Step | -2 Log likelihood | Cox & Snell R Square | Nagelkerke R Square |
| 1 | 34.820^a^ | .537 | .727 |

| a. Estimation terminated at iteration number 20 because maximum iterations has been reached. Final solution cannot be found. |
| --- |

| **Hosmer and Lemeshow Test** | | | |
| --- | --- | --- | --- |
| Step | Chi-square | df | Sig. |
| 1 | 4.719 | 8 | .787 |

| **Contingency Table for Hosmer and Lemeshow Test** | | | | | | |
| --- | --- | --- | --- | --- | --- | --- |
|  | | Newly diagnosed Type 1DM = With DKA | | Newly diagnosed Type 1DM = Without DKA | | Total |
|  |  | Observed | Expected | Observed | Expected |  |
| Step 1 | 1 | 6 | 5.993 | 0 | .007 | 6 |
|  | 2 | 6 | 5.965 | 0 | .035 | 6 |
|  | 3 | 6 | 5.915 | 0 | .085 | 6 |
|  | 4 | 5 | 5.697 | 1 | .303 | 6 |
|  | 5 | 6 | 5.158 | 0 | .842 | 6 |
|  | 6 | 4 | 3.755 | 2 | 2.245 | 6 |
|  | 7 | 2 | 3.030 | 5 | 3.970 | 7 |
|  | 8 | 2 | 1.106 | 5 | 5.894 | 7 |
|  | 9 | 0 | .354 | 6 | 5.646 | 6 |
|  | 10 | 0 | .028 | 5 | 4.972 | 5 |

| **Classification Table^a^** | | | | | |
| --- | --- | --- | --- | --- | --- |
|  | Observed | | Predicted | | |
|  |  |  | Newly diagnosed Type 1DM | | Percentage Correct |
|  |  |  | With DKA | Without DKA |  |
| Step 1 | Newly diagnosed Type 1DM | With DKA | 33 | 4 | 89.2 |
|  |  | Without DKA | 4 | 20 | 83.3 |
|  | Overall Percentage | |  |  | 86.9 |

| a. The cut value is .500 |
| --- |

| **Variables in the Equation** | | | | | | |
| --- | --- | --- | --- | --- | --- | --- |
|  | | B | S.E. | Wald | df | Sig. |
|  |  |  |  |  |  |  |
| Step 1^a^ | infectionbeforedka(1) | 2.459 | 1.105 | 4.953 | 1 | .026 |
|  | educfather |  |  | 7.187 | 4 | .126 |
|  | educfather(1) | -20.334 | 40193.011 | .000 | 1 | 1.000 |
|  | educfather(2) | -22.034 | 40193.011 | .000 | 1 | 1.000 |
|  | educfather(3) | -22.147 | 40193.011 | .000 | 1 | 1.000 |
|  | educfather(4) | -18.677 | 40193.011 | .000 | 1 | 1.000 |
|  | familyincome | .387 | .395 | .959 | 1 | .328 |
|  | firstdegrerelative(1) | -2.048 | .990 | 4.282 | 1 | .039 |
|  | parenetknowsignsymp(1) | -2.655 | 1.109 | 5.727 | 1 | .017 |
|  | Constant | 19.244 | 40193.011 | .000 | 1 | 1.000 |

| **Variables in the Equation** | | | | |
| --- | --- | --- | --- | --- |
|  | | Exp(B) | 95% C.I.for EXP(B) | |
|  |  |  | Lower | Upper |
| Step 1^a^ | infectionbeforedka(1) | 11.691 | 1.341 | 101.925 |
|  | educfather |  |  |  |
|  | educfather(1) | .000 | .000 | . |
|  | educfather(2) | .000 | .000 | . |
|  | educfather(3) | .000 | .000 | . |
|  | educfather(4) | .000 | .000 | . |
|  | familyincome | 1.473 | .679 | 3.195 |
|  | firstdegrerelative(1) | .129 | .019 | .897 |
|  | parenetknowsignsymp(1) | .070 | .008 | .618 |
|  | Constant | 227870395.298 |  |  |

| a. Variable(s) entered on step 1: infectionbeforedka, educfather, familyincome, firstdegrerelative, parenetknowsignsymp. |
| --- |

| **Correlations** | | | | | | | | | | | | | | | |
| --- | --- | --- | --- | --- | --- | --- | --- | --- | --- | --- | --- | --- | --- | --- | --- |
|  | | The child have sign and symptom of DM 2 week before the onset of dka | Which symptom of dm child presented with | Child have frist degre relative with dm | The child parent know sign and symptom of DM/DKA | Child have sign and symptom of infection two week before onset of dka | Age of child | Sex of child | Educational level of mother | Educational level of father | Marital status of parents | Average family incomeper month | Symptom of infection child present with | Symptom DKA the child present with | Newly diagnosed Type 1DM |
| The child have sign and symptom of DM 2 week before the onset of dka | Pearson Correlation | 1 | .^a^ | -.103 | .173 | -.109 | .232 | .051 | .025 | .021 | .234 | -.156 | -.360 | .112 | -.240 |
|  | Sig. (2-tailed) |  | .000 | .428 | .182 | .404 | .072 | .695 | .846 | .874 | .070 | .229 | .109 | .511 | .062 |
|  | N | 61 | 52 | 61 | 61 | 61 | 61 | 61 | 61 | 61 | 61 | 61 | 21 | 37 | 61 |
| Which symptom of dm child presented with | Pearson Correlation | .^a^ | 1 | .306^*^ | -.009 | -.009 | -.031 | -.009 | -.182 | -.140 | -.173 | -.089 | -.335 | .305 | -.058 |
|  | Sig. (2-tailed) | .000 |  | .027 | .952 | .948 | .827 | .948 | .198 | .321 | .221 | .531 | .205 | .108 | .682 |
|  | N | 52 | 52 | 52 | 52 | 52 | 52 | 52 | 52 | 52 | 52 | 52 | 16 | 29 | 52 |
| Child have frist degre relative with dm | Pearson Correlation | -.103 | .306^*^ | 1 | .433^**^ | -.037 | -.195 | .152 | -.173 | -.203 | .055 | -.109 | .360 | .169 | -.367^**^ |
|  | Sig. (2-tailed) | .428 | .027 |  | .000 | .777 | .131 | .241 | .184 | .117 | .672 | .402 | .109 | .318 | .004 |
|  | N | 61 | 52 | 61 | 61 | 61 | 61 | 61 | 61 | 61 | 61 | 61 | 21 | 37 | 61 |
| The child parent know sign and symptom of DM/DKA | Pearson Correlation | .173 | -.009 | .433^**^ | 1 | -.204 | .050 | .270^*^ | -.174 | -.238 | .036 | -.366^**^ | .112 | .004 | -.540^**^ |
|  | Sig. (2-tailed) | .182 | .952 | .000 |  | .115 | .701 | .035 | .179 | .065 | .785 | .004 | .630 | .981 | .000 |
|  | N | 61 | 52 | 61 | 61 | 61 | 61 | 61 | 61 | 61 | 61 | 61 | 21 | 37 | 61 |
| Child have sign and symptom of infection two week before onset of dka | Pearson Correlation | -.109 | -.009 | -.037 | -.204 | 1 | .070 | .120 | .074 | .081 | .123 | .257^*^ | .^a^ | .329^*^ | .355^**^ |
|  | Sig. (2-tailed) | .404 | .948 | .777 | .115 |  | .593 | .356 | .569 | .537 | .345 | .046 | .000 | .047 | .005 |
|  | N | 61 | 52 | 61 | 61 | 61 | 61 | 61 | 61 | 61 | 61 | 61 | 21 | 37 | 61 |
| Age of child | Pearson Correlation | .232 | -.031 | -.195 | .050 | .070 | 1 | .111 | .101 | -.143 | .273^*^ | .068 | -.381 | .290 | .024 |
|  | Sig. (2-tailed) | .072 | .827 | .131 | .701 | .593 |  | .394 | .438 | .273 | .033 | .600 | .088 | .081 | .852 |
|  | N | 61 | 52 | 61 | 61 | 61 | 61 | 61 | 61 | 61 | 61 | 61 | 21 | 37 | 61 |
| Sex of child | Pearson Correlation | .051 | -.009 | .152 | .270^*^ | .120 | .111 | 1 | -.093 | -.080 | -.031 | .099 | -.077 | .140 | -.107 |
|  | Sig. (2-tailed) | .695 | .948 | .241 | .035 | .356 | .394 |  | .475 | .542 | .812 | .447 | .740 | .407 | .412 |
|  | N | 61 | 52 | 61 | 61 | 61 | 61 | 61 | 61 | 61 | 61 | 61 | 21 | 37 | 61 |
| Educational level of mother | Pearson Correlation | .025 | -.182 | -.173 | -.174 | .074 | .101 | -.093 | 1 | .568^**^ | .002 | .460^**^ | -.142 | .083 | .126 |
|  | Sig. (2-tailed) | .846 | .198 | .184 | .179 | .569 | .438 | .475 |  | .000 | .989 | .000 | .538 | .625 | .332 |
|  | N | 61 | 52 | 61 | 61 | 61 | 61 | 61 | 61 | 61 | 61 | 61 | 21 | 37 | 61 |
| Educational level of father | Pearson Correlation | .021 | -.140 | -.203 | -.238 | .081 | -.143 | -.080 | .568^**^ | 1 | -.044 | .559^**^ | -.171 | -.021 | .346^**^ |
|  | Sig. (2-tailed) | .874 | .321 | .117 | .065 | .537 | .273 | .542 | .000 |  | .739 | .000 | .459 | .903 | .006 |
|  | N | 61 | 52 | 61 | 61 | 61 | 61 | 61 | 61 | 61 | 61 | 61 | 21 | 37 | 61 |
| Marital status of parents | Pearson Correlation | .234 | -.173 | .055 | .036 | .123 | .273^*^ | -.031 | .002 | -.044 | 1 | .039 | -.165 | .272 | .031 |
|  | Sig. (2-tailed) | .070 | .221 | .672 | .785 | .345 | .033 | .812 | .989 | .739 |  | .768 | .474 | .103 | .812 |
|  | N | 61 | 52 | 61 | 61 | 61 | 61 | 61 | 61 | 61 | 61 | 61 | 21 | 37 | 61 |
| Average family incomeper month | Pearson Correlation | -.156 | -.089 | -.109 | -.366^**^ | .257^*^ | .068 | .099 | .460^**^ | .559^**^ | .039 | 1 | -.128 | .087 | .487^**^ |
|  | Sig. (2-tailed) | .229 | .531 | .402 | .004 | .046 | .600 | .447 | .000 | .000 | .768 |  | .580 | .608 | .000 |
|  | N | 61 | 52 | 61 | 61 | 61 | 61 | 61 | 61 | 61 | 61 | 61 | 21 | 37 | 61 |
| Symptom of infection child present with | Pearson Correlation | -.360 | -.335 | .360 | .112 | .^a^ | -.381 | -.077 | -.142 | -.171 | -.165 | -.128 | 1 | -.269 | .^a^ |
|  | Sig. (2-tailed) | .109 | .205 | .109 | .630 | .000 | .088 | .740 | .538 | .459 | .474 | .580 |  | .238 | .000 |
|  | N | 21 | 16 | 21 | 21 | 21 | 21 | 21 | 21 | 21 | 21 | 21 | 21 | 21 | 21 |
| Symptom DKA the child present with | Pearson Correlation | .112 | .305 | .169 | .004 | .329^*^ | .290 | .140 | .083 | -.021 | .272 | .087 | -.269 | 1 | .^a^ |
|  | Sig. (2-tailed) | .511 | .108 | .318 | .981 | .047 | .081 | .407 | .625 | .903 | .103 | .608 | .238 |  | .000 |
|  | N | 37 | 29 | 37 | 37 | 37 | 37 | 37 | 37 | 37 | 37 | 37 | 21 | 37 | 37 |
| Newly diagnosed Type 1DM | Pearson Correlation | -.240 | -.058 | -.367^**^ | -.540^**^ | .355^**^ | .024 | -.107 | .126 | .346^**^ | .031 | .487^**^ | .^a^ | .^a^ | 1 |
|  | Sig. (2-tailed) | .062 | .682 | .004 | .000 | .005 | .852 | .412 | .332 | .006 | .812 | .000 | .000 | .000 |  |
|  | N | 61 | 52 | 61 | 61 | 61 | 61 | 61 | 61 | 61 | 61 | 61 | 21 | 37 | 61 |
| *. Correlation is significant at the 0.05 level (2-tailed). | | | | | | | | | | | | | | | |
| **. Correlation is significant at the 0.01 level (2-tailed). | | | | | | | | | | | | | | | |
| a. Cannot be computed because at least one of the variables is constant. | | | | | | | | | | | | | | | |
